# *Candida albicans* Induces Selective Development of Macrophages and Monocyte Derived Dendritic Cells by a TLR2 Dependent Signalling

**DOI:** 10.1371/journal.pone.0024761

**Published:** 2011-09-15

**Authors:** Alberto Yáñez, Javier Megías, José-Enrique O'Connor, Daniel Gozalbo, M. Luisa Gil

**Affiliations:** 1 Departamento de Microbiología y Ecología, Universitat de València, Burjassot, Spain; 2 Laboratorio de Citómica, Unidad Mixta CIPF-UVEG, Centro de Investigación “Príncipe Felipe”, Valencia, Spain; Worcester Polytechnic Institute, United States of America

## Abstract

As TLRs are expressed by haematopoietic stem and progenitor cells (HSPCs), these receptors may play a role in haematopoiesis in response to pathogens during infection. We have previously demonstrated that in *in vitro* defined conditions inactivated yeasts and hyphae of *Candida albicans* induce HSPCs proliferation and differentiation towards the myeloid lineage by a TLR2/MyD88 dependent pathway. In this work, we showed that *C. albicans* invasive infection with a low virulence strain results in a rapid expansion of HSPCs (identified as LKS cells: Lin^−^ c-Kit^+^ Sca-1^+^ IL-7Rα^−^), that reach the maximum at day 3 post-infection. This *in vivo* expansion of LKS cells in TLR2^−/−^ mice was delayed until day 7 post- infection. Candidiasis was, as expected, accompanied by an increase in granulopoiesis and decreased lymphopoiesis in the bone marrow. These changes were more pronounced in TLR2^−/−^ mice correlating with their higher fungal burden. Accordingly, emigration of Ly6C^high^ monocytes and neutrophils to spleen was increased in TLR2^−/−^ mice, although the increase in macrophages and inflammatory macrophages was completely dependent on TLR2. Similarly, we detected for the first time, in the spleen of *C. albicans* infected control mice, a newly generated population of dendritic cells that have the phenotype of monocyte derived dendritic cells (moDCs) that were not generated in TLR2^−/−^ infected mice. In addition, *C. albicans* signalling through TLR2/MyD88 and Dectin-1 promotes *in vitro* the differentiation of Lin^−^ cells towards moDCs that secrete TNF-α and are able to kill the microorganism. Therefore, our results indicate that during infection *C. albicans* can directly stimulate progenitor cells through TLR2 and Dectin-1 to generate newly formed inflammatory macrophages and moDCs that may fulfill an essential role in defense mechanisms against the pathogen.

## Introduction

Toll-like receptors (TLRs) constitute a family of pattern-recognition receptors (PRRs) that recognize molecular signatures of microbial pathogens (microbial associated molecular patterns: MAMPs) and function as sensors of infection that induce the activation of the innate immune responses as well as the subsequent development of adaptive immune responses [1]. Recent findings suggest that TLRs may play a role in haematopoiesis during infection [2]. Murine haematopoietic stem cells (HSCs) and their progeny express TLRs, and upon *in vitro* exposure to soluble TLR ligands are stimulated to enter cell cycle and acquire lineage markers [3]; signalling through TLR7/8 induces the differentiation of human bone marrow CD34^+^ progenitor cells along the myeloid lineage [4], and the TLR1/2 agonist Pam_3_CSK_4_ instructs commitment of human HSCs to a myeloid cell fate [5]. In addition, this new mechanism, that represents a potential means for pathogen products to signal the rapid generation of innate immune cells, has been explored in some *in vivo* infectious models showing that: (i) murine lymphoid precursors are directed to produce dendritic cells as a result of TLR9 ligation during herpes infection [6], (ii) vaccinia virus infection results in the expansion of HSCs in a myeloid differentiation factor 88 (MyD88)-dependent manner [7] and (iii) TLR-mediated signals play an essential role in the monocyte expansion during *Listeria monocytogenes* systemic infection [8].

*Candida albicans* is the microorganism most frequently causing opportunistic fungal infections. Clinical manifestations range from mucosal candidiasis to life-threatening invasive infections, whose frequency has increased in the last decades as a result of an expanding immunocompromised population. Resistance to candidiasis requires the coordinated action of innate and adaptive immune defenses [9], [10]. Phagocytes, such as neutrophils and macrophages, are crucial to these processes since they can eliminate the pathogen via phagocytosis. Furthermore, macrophage and dendritic cell activation leads to the release of several key mediators such as proinflammatory cytokines, which are important for protecting the host against disseminated candidasis, and for inducing a T helper type 1 (Th1) immune response that activates fungicidal effector mechanisms [9], [10]. Phagocytotic cells recognize the pathogen through a variety of pattern recognition receptors (PRRs), including TLRs and Dectin-1 [11], [12]. We have previously shown that TLR2 is the most important TLR involved in interaction with *C. albicans*, both yeasts and hyphae, triggering cytokine secretion through the MyD88 signalling pathway [13], [14], [15]. Other authors have also described the involvement of TLR4 in *C. albicans* recognition, and it is now accepted that both TLR2, which forms TLR2/TLR6 heterodimers, and TLR4 are the main TLRs involved in the signalling induced by *C. albicans* [12], [16], [17]. Dectin-1 is a phagocytic receptor that recognizes β-glucan in the cell wall of *C. albicans*, and also collaborates with TLR2 in the elicitation of proinflammatory cytokines [16].

Recently, we have demonstrated that *C. albicans* induces *in vitro* the proliferation of HSPCs and also their differentiation towards the myeloid lineage. This response requires signalling through TLR2/MyD88, and give rise to functional phagocytes that are able to internalize yeasts and secrete proinflammatory cytokines [18], [19]. Here we show that the bone marrow LKS (Lin^−^ c-Kit^+^ Sca-1^+^ IL-7Rα^−^) cell population is rapidly expanded following *C. albicans* fungemia in a TLR2-dependent manner. This was associated with a drastic decrease in B cells and increased amounts of neutrophils and monocytes; these changes were more pronounced in TLR2^−/−^ mice although the percentages of pre-DCs and macrophages were lower in the bone marrow of TLR2^−/−^ infected mice. Similarly, the expansion of inflammatory macrophages and monocyte-derived dendritic cells (moDCs) in the spleen was completely dependent on TLR2. In addition, *C. albicans* signalling through TLR2/MyD88 and Dectin-1 promotes *in vitro* the differentiation of Lin^−^ progenitor cells towards moDCs that secrete proinflamatory cytokines and are able to kill the microorganism. This is the first identification of moDCs in *C. albicans* infected tissues, and our results suggests that *C. albicans* can directly stimulate progenitor cells through TLR2/MyD88 and Dectin-1 to generate newly formed inflammatory macrophages and moDCs during infection that may fulfill an essential role in defense mechanisms against the pathogen.

## Results

### Increased fungal burden in *C. albicans*-infected TLR2^−/−^ mice

**Figure 1:**
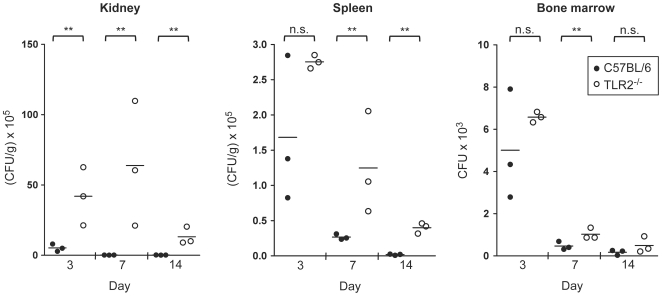
Fungal burden in the kidney, spleen and bone marrow of *C. albicans* infected mice. C57BL/6 (n = 20) and TLR2^−/−^ mice (n = 20) were injected intravenously with 1.5×10^6^ yeasts of *C. albicans* PCA2 per mouse. Three mice of each group were killed at days 3, 7 and 14 post-infection, to assess the outgrowth of the yeasts in the kidneys and spleen (expressed as CFUs per gram of tissue) and in the bone marrow (expressed as CFUs per total bone marrow from femurs and tibias from one animal). Data represent single values of each sample (n = 3), from one representative experiment of two. Mean values are also indicated. ** *P*<0.01 with respect to the wild-type mice.

We have previously reported that *in vitro*, inactivated yeasts and hyphae of *C. albicans* induce the proliferation of murine HSPCs as well as their differentiation to myeloid cells, through a TLR2/MyD88 dependent pathway [18], [19]. The observed *in vitro* effect of *C. albicans* on HSPCs activation may be of biological relevance *in vivo*. During invasive candidiasis fungal cells or soluble MAMPs (microorganism-associated molecular patterns) may rapidly reach the bone marrow cavity and engage the receptors of stem and progenitor cells. Therefore, in order to determine whether TLR2 may be involved in the haematopoietic process during candidasis, TLR2^−/−^ and control mice were infected with *C. albicans*. Because of the increased susceptibility of TLR2^−/−^ mice to systemic candidiasis [20] the low-virulence PCA2 strain was chosen for the infection, in order to avoid mice mortality and severe differences in fungal burden between control and knockout mice; this strain has been widely used to study the immune response to *C. albicans* infections [21], [22], [23], [24]. During the infection, we first tested the fungal burden in the primary and secondary lymphoid organs, the bone marrow and spleen, respectively, as well as in the kidney, the target organ in this invasive model of candidiasis (Figure 1). When TLR2^−/−^ and C57BL/6 mice were intravenously infected with 1.5×10^6^ yeasts, it was found that fungal cells are able to reach the bone marrow and the amount of yeasts recovered from this site at day 3 post-infection was roughly 6000 colony-forming units (CFUs) per animal. No significant differences in the fungal burden between control and TLR2^−/−^ mice were found in the bone marrow and spleen 3 days post-infection. However, the actual fungal burden in the kidney was significantly higher in TLR2^−/−^ mice, compared with control mice, at days 3, 7 and 14 post-infection, according with the higher susceptibility of TLR2^−/−^ mice to invasive candidiasis previously described by our group [15], [17], [20]. Similarly, the CFUs in the spleen at days 7 and 14 post-infection, as well as in the bone marrow at day 7 post-infection, were significantly higher in TLR2^−/−^ than in control mice. Moreover, TLR2^−/−^ mice consistently appeared sicker (hunched back, ruffled fur and slow in movement) than control mice, and two out of 20 infected TLR2^−/−^ mice (10%) succumbed between day 4 and 6 after infection, while none of control mice died. Interestingly, both groups of mice were able to reduce fungal burden throughout the infection indicating that these mice were able to overcome invasion of tissues by the low-virulence strain.

### *C. albicans* infection induces early TLR2-dependent expansion of HSPCs

**Figure 2:**
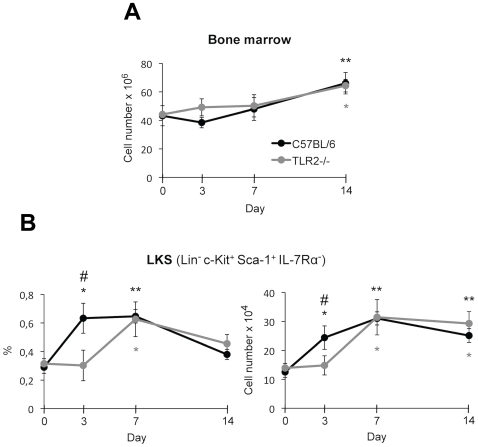
LKS cells in bone marrow of *C. albicans* infected mice. C57BL/6 and TLR2^−/−^ mice (n = 20) were injected intravenously with 1.5×10^6^ yeasts of *C. albicans* PCA2 per mouse. Three mice of each group were killed at days 3, 7 and 14 post-infection, and bone marrow cells were isolated, erythrocytes were lysed and cells were microscopically counted (total nucleated bone marrow cells) (A), labelled with various combinations of antibodies and analyzed by flow cytometry to assess the changes in LKS population (expressed both as percentage of total bone marrow cells and as total LKS cells (B). Data represent means ± SD (n = 3), from one representative experiment of two. * *P*<0.05, ** *P*<0.01 when each day is compared with day 0 (uninfected mice). # *P*<0.05 when TLR2^−/−^ mice are compared with the wild-type mice.

All leucocytes arise from a common ancestor, the haematopoietic stem cells (HSCs), which are self-renewing, and through asymmetric division give rise to more differentiated non-renewing multipotent progenitors (MPPs). MPPs gradually lose developmental plasticity and produce committed lineages known as common myeloid progenitors (CMPs) and common lymphoid progenitors (CLPs). Nowadays, the prevailing definition for murine HSPCs is the LKS (Lin^−^ c-Kit^+^ Sca-1^+^ IL-7Rα^−^) population, which contains a variety of transitional intermediates between long-term repopulating HSCs, and oligopotent progenitors (long-term repopulating HSCs, short-term repopulating HSCs and MPPs) [25]. Thus, to examine changes in the bone marrow HSPCs following systemic infection with *C. albicans*, bone marrow cells were analyzed by flow cytometry on the basis of their phenotypic surface markers, at several days post-infection. As shown in Figure 2B, both the proportion and the amount of LKS cells in the bone marrow of control mice were rapidly increased, reaching the maximum at day 3 and day 7 post-infection. The increased amount of LKS cells was sustained until day 14, although the percentage of LKS population returned to close to normal at day 14, since the total number of bone marrow cells was significantly increased at that day (Figure 2A). The early increase of LKS cells observed at day 3 was dependent on TLR2, as it did not occur in the bone marrow of TLR2^−/−^ mice until day 7.

### Candidiasis induces bone marrow remodelling by TLR2 dependent and independent mechanisms

**Figure 3:**
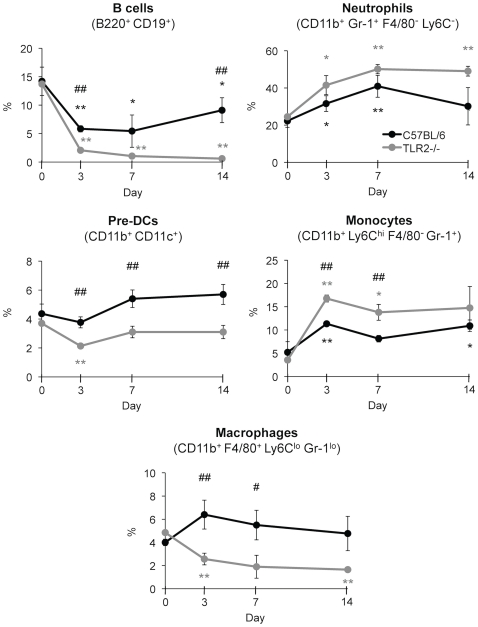
Bone marrow remodelling in *C. albicans* infected mice. C57BL/6 and TLR2^−/−^ mice (n = 20) were injected intravenously with 1.5×10^6^ yeasts of *C. albicans* PCA2 per mouse. Three mice of each group were killed at days 3, 7 and 14 post-infection, and bone marrow cells were isolated, erythrocytes were lysed and cells were microscopically counted, labelled with various combinations of antibodies and analyzed by flow cytometry to assess the changes in B cells, neutrophils, pre-DCs, monocytes and macrophages, expressed as percentage of total bone marrow cells (see Figure 2A). Data represent means ± SD (n = 3), from one representative experiment of two. * *P*<0.05, ** *P*<0.01 when each day is compared with day 0 (uninfected mice). # *P*<0.05, ## *P*<0.01 when TLR2^−/−^ mice are compared with the wild-type mice.

Next, we wondered whether *C. albicans* infection may alter mature cell production in the bone marrow through a mechanism involving TLR2. At days 3, 7 and 14 post-infection, the frequency of bone marrow B cells, neutrophils, pre-DCs, Ly6C^high^ monocytes and macrophages was analyzed by flow cytometry multifluorescence (Figure 3). According to previous reports [19], [26], we observed that during *C. albicans* infection there is an increase in granulopoiesis, as the percentage of neutrophils in the bone marrow of control mice increased during the first week, whereas lymphopoiesis was inhibited, as the amount of B cells decreased dramatically. In addition, the frequency of Ly6C^high^ monocytes was also rapidly increased at day 3 and sustained until day 14, probably due to the equilibrium between enhanced production and continuous emigration to peripheral infected sites. The percentage of pre-DCs was slightly increased during infection. It should be noted that percentage of cell populations correlates with absolute cell number, as no differences were found for total bone marrow cells in both control and TLR2^−/−^ mice.

The increased percentage of neutrophils and Ly6C^high^ monocytes and the decrease in B cells was more pronounced in TLR2^−/−^ mice. The frequency of pre-DCs in TLR2^−/−^ mice was sustained after an initial decrease at day 3, and differences with control mice were statistically significant. Interestingly, the frequency of macrophages clearly increases in control mice at day 3 post-infection whereas this cell type decreases in the bone marrow of TLR2^−/−^ mice despite no significant statistical differences were found in the fungal burden of both groups of mice in the bone marrow at day 3. This result clearly indicates that *C. albicans* infection induces early TLR2-dependent expansion of macrophages.

### Infection-induced macrophage and dendritic cell expansion in the spleen involves TLR2 signalling

**Figure 4:**
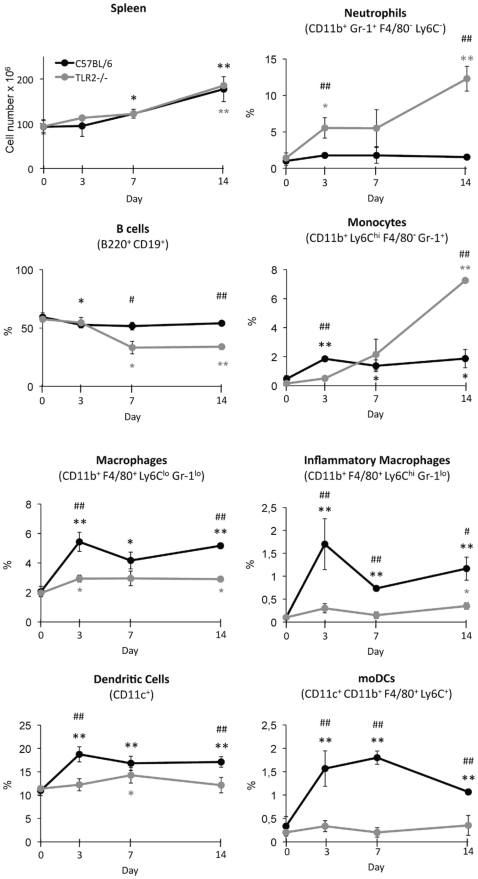
Macrophage and dendritic cell expansion in the spleen of *C. albicans* infected mice. C57BL/6 and TLR2^−/−^ mice (n = 20) were injected intravenously with 1.5×10^6^ yeasts of *C. albicans* PCA2 per mouse. Three mice of each group were killed at days 3, 7 and 14 post-infection, and total spleen cells were obtained by collagenase D treatment of the spleens, erythrocytes were lysed and cells were microscopically counted, labelled with various combinations of antibodies and analyzed by flow cytometry to assess the changes in neutrophils, B cells, monocytes, macrophages, inflammatory macrophages, DCs and moDCs. Percentages of each cell type are referred to the total cell number in the spleen. Data represent means ± SD, from one representative experiment of two. * *P*<0.05, ** *P*<0.01 when each day is compared with day 0 (uninfected mice). # *P*<0.05, ## *P*<0.01 when TLR2^−/−^ mice are compared with the wild-type mice.

As neutrophils and monocytes are continuously emigrating from bone marrow to peripheral tissues, the measure of bone marrow cellularity may underestimate newly generated cells. Therefore, the frequency of different cell types in the spleen was analyzed (Figure 4). As above mentioned, the percentage of cell types correlates with absolute cell number, as no differences were found for total spleen cells in both control and TLR2^−/−^ mice. A marked neutrophil recruitment was observed to the spleen of TLR2^−/−^ infected mice but not in control mice, particularly at day 14 post-infection, correlating with the higher fungal burden in knockout mice. Similarly, at day 14 post- infection, an increased recruitment of Ly6C^high^ monocytes was observed to the spleen of TLR2^−/−^ infected mice, whereas control mice showed a discrete recruitment of monocytes by day 3. However, the increase of macrophages and total DCs, as well as the new generation of inflammatory macrophages observed early after infection, in wild-type mice, were absent or significantly reduced in TLR2^−/−^ mice. Interestingly, a population of CD11c^+^ cells that were CD11b^+^ but also F4/80^+^ and Ly6C^high^ was identified in the spleen of control infected mice; this population of cells with the described phenotype of the moDCs [27] was not detected in TLR2^−/−^ infected mice. This result clearly indicates that *C. albicans* infection induces TLR2-dependent generation of inflammatory macrophages and moDCs.

### *C. albicans* drives *in vitro* TLR2/Dectin-1-dependent production of monocyte derived dendritic cells from lineage negative progenitors

**Figure 5:**
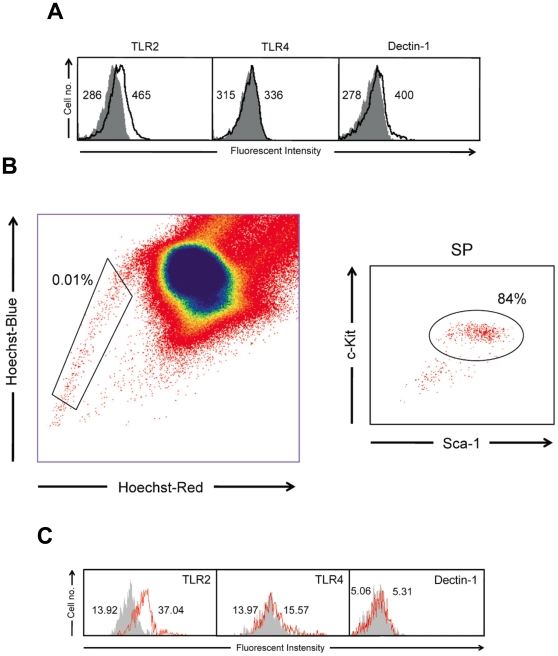
TLR2, TLR4 and Dectin-1 expression on Lin^−^ progenitor cells and LT-HSCs (SP). (A) Fluorescence mean intensity of TLR2, TLR4 and Dectin-1 staining with the specific monoclonal antibodies (number on the right) and their respective isotype control (number on the left) measured by flow cytometry, in Lin^−^ progenitor cells. (B) Identification of SP population. Hoeschst Red and Hoeschst Blue parameters were examined allowing the SP population to be identified and gated; the percentage of SP cells measured in the total nucleated viable cells is indicated. The SP cells were then displayed for their Sca-1 versus c-Kit plot: the gate indicates the percentage of double positive population. (C) Fluorescence mean intensity of TLR2, TLR4 and Dectin-1 staining with the specific monoclonal antibodies (number on the right) and their respective isotype control (number on the left) measured by flow cytometry, in SP gated cells. The results shown represent the data from one representative experiment of two.

First, the expression of the most important TLRs involved in *C. albicans* recognition was determined in Lin^−^ progenitor cells (Figure 5A). Results showed a high expression of TLR2, and a very little expression of TLR4 in these cells, in agreement with a previous publication [3]. As mature cells also recognize *C. albicans* by PRRs other than TLRs, such as Dectin-1, the expression of this receptor was studied in Lin^−^ cells. Results showed that Lin^−^ progenitor cells actually express detectable levels of Dectin-1 (Figure 5A). This result prompted us to investigate whether HSCs would also express Dectin-1. Hoechst 33342 staining of total bone marrow cells was used to identify bona fide HSCs, the so-called side population (SP) that contains all the long-term HSC activity. The dye is retained at low levels in HSCs because of their ability to efflux it, and therefore this distinctive staining pattern is easily observed by flow cytometry. When the staining is correctly performed, the frequency of SP cells should always remain within 0.01–0.03% of bone marrow cells (excluding dead cells and red blood cells) and ∼85% of SP should be LKS [28]. As shown in Figure 5B our SP identification fulfill these conditions, and as shown in Figure 5C, these cells express TLR2 and TLR4 in a minor extent, but not Dectin-1. These results clearly indicate that while TLRs are expressed by the most primitive stem cells, the expression of Dectin-1 is acquired by progenitors in a more advanced state of differentiation.

**Figure 6:**
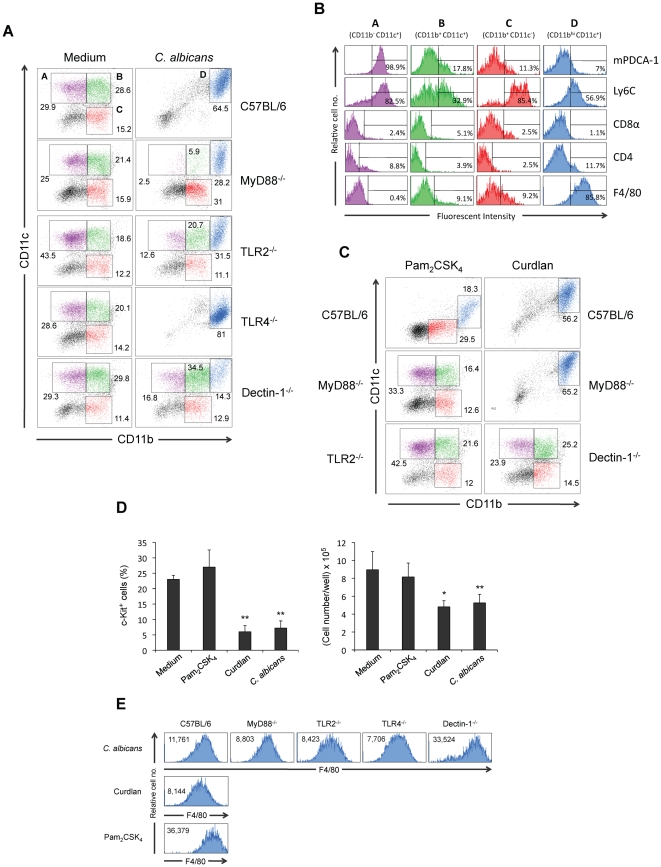
*In vitro* differentiation of Lin^−^ progenitor cells in response to *C. albicans*. Lin^−^ progenitor cells (400,000 cells/well in 0.5 ml) from C57BL/6, MyD88^−/−^, TLR2^−/−^, TLR4^−/−^ and Dectin-1^−/−^ mice were cultured in the presence of SCF, FL and IL-7 with medium alone (Medium), Pam_2_CSK_4_ (0.125 µg/ml), Curdlan (100 µg/ml) or inactivated yeasts of *C. albicans* ATCC 26555 [120 µg (dry weight) of cells/ml] for 7 days. (A) and (C) Cells were labelled with anti-CD11b and anti-CD11c antibodies and analyzed by flow cytometry. Cells were gated as population A (CD11b^−^ CD11c^+^, violet gate), population B (CD11b^+^ CD11c^+^, green gate), population C (CD11b^+^ CD11c^−^, red gate) and population D (CD11b^high^ CD11c^+^, blue gate). (B) Expression of mPDCA-1, Ly6C, CD8α, CD4 and F4/80 were analyzed in the gated populations; the percentage of positive cells is indicated. (D) The expression of c-Kit was analyzed by flow cytometry and indicated as % of positive cells, whereas the total number of cells was determined microscopically. (E) Expression of F4/80 was analyzed in CD11b^high^ CD11c^+^ and CD11b^high^ CD11c^low^ populations (blue gates). All cell populations analyzed are F4/80 positive but differ in the mean fluorescence intensity, as indicated Data represent means ± SD, from one representative experiment of three. * *P*<0.05, ** *P*<0.01 with respect to cells incubated with medium alone (Medium).

**Figure 7:**
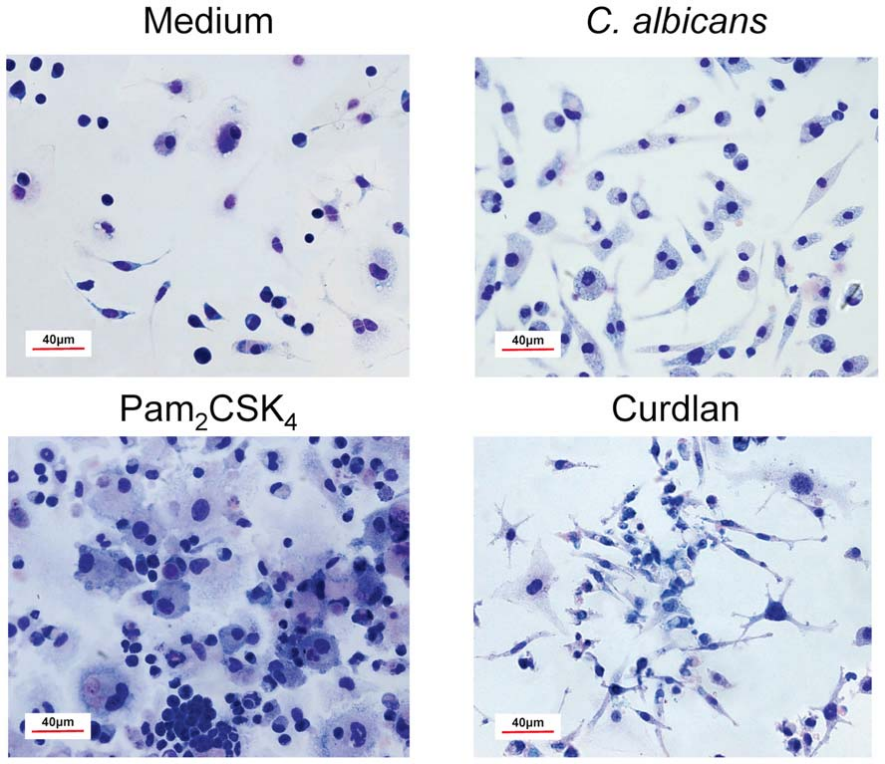
Morphology of Lin^−^ progenitor cells differentiated *in vitro*. Lin^−^ progenitor cells from C57BL/6 mice were cultured in the presence of SCF, FL and IL-7 with medium alone (Medium), Pam_2_CSK_4_ (0.125 µg/ml), Curdlan (100 µg/ml) or inactivated yeasts of *C. albicans* ATCC 26555 [120 µg (dry weight) of cells/ml] for 7 days. Cells were then stained with Rapid Panoptic.

Next, we determined the *in vitro* effect of fungal ligands on differentiation of Lin^−^ progenitor cells. These cells from control mice, cultured for 7 days in complete cell culture medium containing stem cell factor (SCF), Flt-3 ligand (FL) and IL-7, differentiate to a mixed population of cells (Figures 6A and 7). These differentiated cells did not express the CD19 lymphoid marker (data not shown), indicating that in these conditions B cells were not produced. Analysis of the expression of CD11b and CD11c allowed the identification of 3 different cell populations (Figure 6A and B): (i) population A (CD11b^−^ CD11c^+^) that also express mPDCA-1 and Ly6C, and therefore correspond to plasmacytoid DCs (pDCs), (ii) population B (CD11b^+^ CD11c^+^) that correspond to pre-DCs or classical DCs double negative for CD4 and CD8α, and (iii) population C (CD11b^+^ CD11c^−^) that correspond to monocytes Ly6C^high^. When Lin^−^ progenitor cells were cultured in the same conditions but in the presence of fungal yeasts the differentiation pattern was completely different (Figure 6A and 7). Analysis of the expression of CD11b and CD11c identified a major population of cells (64.5%, population D) that was CD11b^high^ CD11c^+^, and also express Ly6C and F4/80, therefore corresponding to the described phenotype for the moDCs. The response to *C. albicans* yeasts was partially dependent on MyD88, TLR2 and Dectin-1 (Figure 6A) as the moDCs represent only 28%, 31% and 14% in the cultures from MyD88^−/−^, TLR2^−/−^ and Dectin-1^−/−^ mice, respectively. In the cultures of TLR4^−/−^ cells, this population represent the majority of cells, indicating that signalling through TLR4 is dispensable for the differentiation to moDCs, although it should be indicated that these cells express lower levels of CD11c than wild-type cells. As expected, Lin^−^ progenitor cells from TLR2^−/−^ mice did not respond to Pam_2_CSK_4_ (a pure TLR2/TLR6 ligand), Lin^−^ progenitor cells from Dectin1^−/−^ mice did not respond to Curdlan (a pure Dectin-1 ligand) and Lin^−^ progenitor cells from MyD88^−/−^ mice responded to Curdlan but did not respond to Pam_2_CSK_4_ (Figure 6C). Interestingly, the response of control Lin^−^ cells to Curdlan was similar to the response to *C. albicans*, whereas the response to Pam_2_CSK_4_ was different. In response to Pam_2_CSK_4,_ a population of CD11b^high^ CD11c^low^ cells, that represent an 18% was identified, as well as a population CD11b^+^ CD11c^−^ of monocytes Ly6C^high^ that represent 29% and a population CD11b^−^ CD11c^−^ that probably corresponds to undifferentiated cells. Accordingly, the percentage of undifferentiated cells (c-Kit^+^) was higher in unstimulated and Pam_2_CSK_4_ treated cultures than in *C. albicans* and Curdlan stimulated cultures and this lower level of differentiation correlates with an increased proliferation measured as total cell number (Figure 6D). The morphology of Lin^−^ progenitor cells differentiated after 7 days in the presence of yeasts, Pam_2_CSK_4_ or Curdlan was observed, and significant differences were found when compared to unstimulated cells (Figure 7). In control cultures, cells showing monocyte, plasmacytoid and dendritic shape were found. As expected, challenge with Pam_2_CSK_4_ induced proliferation of monocytes and macrophages, whereas the morphology of DCs generated in response to Curdlan displayed more dendritic extensions than the DCs generated in response to yeasts. To further characterize the CD11b^high^ CD11c^+^ and CD11b^high^ CD11c^low^ populations, these cells were analyzed for the expression of F4/80 (Figure 6E). Results showed that all these cell populations are F4/80 positive and express similar levels of this marker, except: (i) the CD11b^high^ CD11c^low^ population obtained in response to Pam_2_CSK_4,_ and (ii) the CD11b^high^ CD11c^+^ population obtained from Dectin1^−/−^ cells in response to yeasts, that express higher level, indicating that TLR2 signalling is upregulating the expression of the F4/80 macrophage marker. The morphology of the CD11b^high^ CD11c^low^ population obtained in response to Pam_2_CSK_4,_ as well as the markers expressed, suggest that these cells may correspond to macrophages instead of moDCs. Overall these results suggest that the *C. albicans* induced differentiation of Lin^−^ progenitor cells to moDCs is dependent on both TLR2/MyD88 and Dectin-1, although the role of Dectin-1 signalling seems to be more significant.

### Mature cells produced from lineage negative progenitors in response to *C. albicans* possess anti-fungal functions

**Figure 8:**
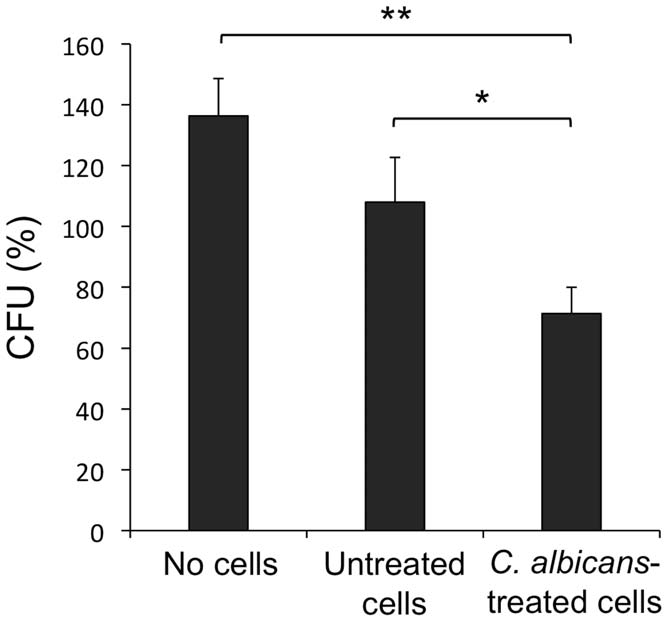
Fungicidal activity of Lin^−^ progenitor cells differentiated *in vitro*. Lin^−^ progenitor cells from C57BL/6 mice were cultured in the presence of SCF, FL and IL-7 with medium alone (Untreated cells) or inactivated yeasts of *C. albicans* ATCC 26555 [1.5 µg (dry weight) of cells/ml] (*C. albicans-*treated cells) for 7 days. Afterwards, cells were plated at a density of 400,000 cells in 200 µl of complete cell culture medium, challenged with viable yeasts of *C. albicans* PCA2 at a 1∶3 ratio (murine cell∶yeast) and incubated for 1 h. *C. albicans* cells were also inoculated in culture medium without murine cells (No cells). After incubation, samples were diluted, plated on Sabouraud dextrose agar and incubated overnight at 37°C; the colonies were counted and expressed as %CFUs as compared with CFUs at time 0 h (100%). Data represent means ± SD from triplicates of one representative experiment of two. ** *P*<0.01 with respect to control, and * *P*<0.05 with respect to medium.

**Figure 9:**
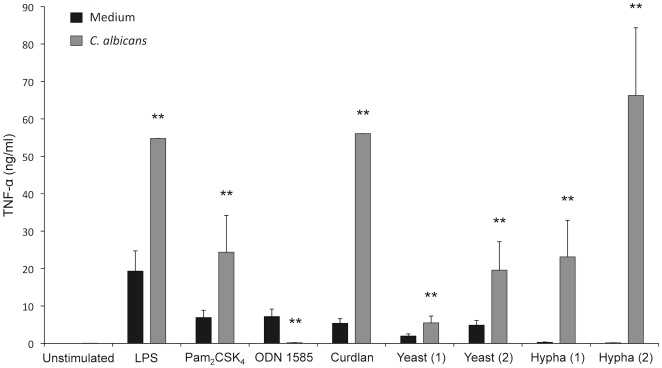
TNF-α production by Lin^−^ progenitor cells differentiated *in vitro*. Lin^−^ progenitor cells from C57BL/6 mice were cultured in the presence of SCF, FL and IL-7 with medium alone (Medium) or inactivated yeasts of *C. albicans* ATCC 26555 [120 µg (dry weight) of cells/ml] (*C. albicans*) for 7 days. Afterwards, cells were plated at a density of 250,000 cells in 200 µl of complete cell culture medium, and challenged for 24 h with ultrapure *E. coli* LPS (0.25 µg/ml), Pam_2_CSK_4_ (4 µg/ml), ODN 1585 (1 µg/ml), Curdlan (10 µg/ml) and two amounts of inactivated *C. albicans* ATCC 26555 yeasts or hyphae: (1) 150 µg (dry weight) of cells/ml or (2) 750 µg (dry weight) of cells/ml. Concentration of TNF-α in cell-free culture supernatants was measured by ELISA. Data represent means ± SD, from triplicates of one representative experiment of three. * *P*<0.05, ** *P*<0.01 with respect to cells incubated with medium alone (Medium).

The above described findings indicate that *C. albicans* yeasts induce the differentiation of Lin^−^ progenitor cells into cells that express the markers of moDCs and that exhibit the morphology of mature cells. These results prompted us to investigate whether these differentiated cells are functional by measuring the ability to kill yeasts cells and to produce proinflammatory cytokines. For this, Lin^−^ progenitor cells from C57BL/6 mice were cultured for 7 days either in presence or absence of *C. albicans* yeasts to induce the differentiation process, and afterwards, cells were challenged either with viable PCA2 yeasts for 1 h, in order to determine the fungicidal activity (Figure 8) or with inactivated yeasts, hyphae or pure ligands for PRRs for 24 h, in order to determine the production of TNF-α (Figure 9).

First, the ability of yeasts to proliferate during the culture in the absence of murine cells was determined, and an increase of roughly 40% in CFUs was found after 1 h incubation (Figure 8, no cells). This proliferation was inhibited by co-culture with murine control cells (Figure 8, untreated cells), whereas cells differentiated in presence of *C. albicans* were able to kill a significant percentage of yeasts (Figure 8, *C. albicans*-treated cells), as CFUs after the co-culture were reduced to 70%.

As shown in Figure 9, production of TNF-α by cells differentiated in response to yeasts was significantly higher than the production by control cells (incubated 7 days in medium without stimuli) after challenge with inactivated yeasts or hyphae, LPS (ligand of TLR4), Pam_2_CSK_4_ (ligand of TLR2/TLR6) or Curdlan (ligand of Dectin-1). Interestingly, control cells secreted more TNF-α in response to ODN 1585 (ligand of TLR9), according with the high expression of this receptor in pDCs [1] which represent roughly one third of total cells (population A, Figure 6A). In addition, the TNF-α production by the *C. albicans* differentiated cells in response to hyphae was higher than in response to yeasts, whereas control cells produced detectable levels of cytokine only in response to yeasts (Figure 9).

These results indicate that Lin^−^ progenitor cells differentiated in the presence of yeasts have acquired fungicidal activity and are able to secrete proinflammatory cytokines.

## Discussion

Under physiological conditions, the process of HSC self-renewal, as well as their conversion into lineage-committed progenitors, is tightly controlled to maintain daily blood cell production. Many cytokines and transcription factors “fine-tune” the proliferation of HSPCs and their differentiation into mature myeloid and lymphoid cells [25]. However, accumulated evidence has shown that haematopoiesis is altered during inflammation and infection, whereby production of phagocytes, particularly granulocytes and monocytes, becomes predominant with inhibition of other lineage (lymphoid and erythroid) development [8], [26], [29]. Additional perspective has come from the discovery that HSPCs express functional TLRs [3]. We previously showed, for the first time, that highly purified stem and progenitor cells directly responded to a microorganism such as *C. albicans* through TLR2/MyD88 under defined conditions of culture [18], [19]. However, the physiologic relevance of this phenomenon has not been explored during experimental candidiasis. The present study shows that systemic infection with a low virulence *C. albicans* strain induces a rapid HSPCs expansion in control mice and that this expansion was delayed in TLR2^−/−^ mice. The LKS expansion after infection has been also described in several models of bacterial and viral infection [7], [30], [31]. However, the contribution of TLR signalling to this phenomenon is still a mater of discussion; Scumpia *et al* [31] described that this expansion following bacterial infection occurs in the absence of TLR signalling, whereas Singh *et al* [7] found that the LKS expansion after vaccinia virus infection is dependent on MyD88, and in this work we have found that the expansion is delayed in the absence of TLR2 signalling during candidiasis. The alteration in the LKS population can be explained by at least two manners: (i) *C. albicans* MAMPs may directly induce stem cell proliferation, as suggested by the *in vitro* results, or alternatively (ii) the LKS expansion could be caused by an indirect effect, due to pathophysiological changes during infection. Both possibilities are not mutually exclusive, and both of them may involve TLR recognition of the pathogen. It is important to notice that the interpretation of the *in vivo* results is difficult as TLR2^−/−^ and particularly MyD88^−/−^ mice are more susceptible to most of the infections; therefore, comparison of haematopoiesis between control and knockout mice during infection may reflect different tissue invasion by the microorganism, and consequently, differences in secretion of cytokines involved in haematopoiesis, which in addition also involve TLR/MyD88 mediated response to the pathogen. In our model of candidiasis, the fungal burden at day 3 post-infection in the bone marrow of control and TLR2^−/−^ mice was similar, but only in control mice the LKS population was expanded, clearly indicating that TLR2 is essential for this rapid HSPCs proliferation, although the precise mechanism has to be still determined.

The cellular composition of the bone marrow and the spleen was impacted by *C. albicans* infection. In the bone marrow, we found the already described increase in neutrophils and decrease in lymphocytes [19], [26], and this effect was more noticeable in TLR2^−/−^ mice correlating with their higher fungal burden. This increased response of TLR2^−/−^ mice may be induced by cytokines produced in the kidney/spleen where there is an increased fungal burden at day 3, and/or to higher levels of circulating fungal components, recognized by PRRs other than TLR2. This result is in agreement with the previous demonstration that granulopoiesis and lymphopoiesis are coupled specifically in the bone marrow by development in a common niche, during infection induced inflammation [29], and with *in vitro* models of B cell differentiation showing that signalling through TLR4 promotes B cell maturation and is inhibited by TLR2 [32]. Moreover, we also found, in the bone marrow, a higher increase of Ly6C^high^ monocytes in TLR2^−/−^ mice than in control mice, while the number of macrophages and pre-DCs was lower in the bone marrow of TLR2^−/−^ mice. Neutrophils and monocytes were recruited to the spleen of TLR2^−/−^ mice, but not to control mice, as the infection by the low virulence PCA2 strain was quickly stopped in wild-type animals. However, the amount of macrophages and total DCs was again lower in the spleen of TLR2^−/−^ mice than in control mice. Interestingly, a newly generated population of inflammatory macrohages and a subset of DCs with the phenotype of moDCs were induced during candidiasis in wild-type mice, but not in TLR2^−/−^ mice, indicating that development of both cell populations is TLR2 dependent. Although monocytes were originally described as precursors of all different subpopulations of macrophages, recent data have demonstrated that monocytes can also differentiate into DCs. Monocytes are the precursors of different subsets of non-lymphoid tissues DCs, such as Langerhans cells. In addition, moDCs that are newly formed during inflammatory reactions appear to have a role in defense mechanisms against pathogens by participating in the induction of both adaptive and innate immune responses [27], [33]. It should be stressed that despite the marked recruitment of Ly6C^high^ monocytes to the spleen of TLR2^−/−^ infected mice, these cells did not differentiate to inflammatory macrophages or moDCs in the absence of TLR2 signalling. These results are in agreement with a report by Krutzik *et al.* [34] showing that TLR activation triggers the rapid differentiation of human monocytes into macrophages and dendritic cells and that this differentiation appears critically to influence effective host defenses in human infectious disease. The newly generated inflammatory macrophages and moDCs in *C. albicans* infected control mice probably contribute to their capability to control the infection at this location.

Our study was performed with the low virulence PCA2 strain in order to avoid mice mortality and severe differences in fungal burden between controls and the most susceptible TLR2^−/−^ mice [14], [15], [17]. Models of infection with a high virulence strain may cause very significant differences in fungal burden and inflammatory cytokine production between control and TLR2^−/−^ mice which make the results very difficult to interpret. In addition, as infection with PCA2 strain has been shown to induce a Th1 response that confers protection to a secondary infection with a virulent strain [22], [23], [24], the use of PCA2 strain seems to be appropriate to study the generation of immune responses in animal models of infection. However, it should be noted that infection with a high virulence strain may induce immune responses different from that generated against PCA2 strain. Preliminary results performed with the high virulence *C. albicans* ATCC 26555 strain showed a different response to that observed to PCA2 strain (not shown). As above mentioned, the very strong difference in tissue invasion, between control and TLR2^−/−^ mice make results difficult to interpret. However, in the spleen of control mice newly generated inflammatory macrophages (1.2%) and moDCs (2.3%) were detected, whereas levels of these cell populations were lower in TLR2^−/−^ mice (0.6% and 0.4%, respectively), suggesting that these cell types are also generated in response to virulent *C. albicans* strains in a TLR2 dependent manner.

In a previous work, we have shown that *C. albicans* drives *in vitro* myeloid differentiation of LKS population, long-term repopulating HSCs, CMPs and granulocyte and macrophage progenitors (GMPs) by a TLR2/MyD88 dependent mechanism; purified cells were cultured in a serum-free, stromal cell-free medium during 3 days, with two cytokines: stem cell factor (SCF) and Flt-3 ligand (FL), as they promote cell viability of these stem and progenitor cells, but induce little differentiation under these well defined conditions [19]. These previous data and the results of the *in vivo* assays, prompted us to investigate the effect of yeast cells on the differentiation of Lin^−^ cells (containing both, stem and all the progenitor cells) in the presence of serum and extending the culture period to 7 days. In this case, IL-7 was added, as well as SCF and FL, to guarantee the survival of CLPs. In these conditions most of the Lin^−^ progenitor cells differentiate to a mixed population of pDCs (30%), classical or pre-DCs (29%) and monocytes Ly6C^high^ (15%). However, when inactivated yeasts of *C. albicans* were added to the cultures, cells differentiated to the phenotype of moDCs (64.5%). The results obtained with Lin^−^ progenitor cells from knockout mice indicate that this differentiation was dependent on both Dectin-1 and TLR2/MyD88, but independent of TLR4. Since the expression of other PRRs (such as manose receptor, galectin-3, etc) in Lin^−^ cells was not determined, and only single knockout mice for TLR2 or Dectin-1 were analyzed, the involvement of other PRRs in the differentiation process can not be completely ruled out. The differentiation generated in response to Curdlan (a pure Dectin-1 ligand) was more similar to the response to *C. albicans* than to the response to Pam_2_CSK_4_ (a pure TLR2/TLR6 ligand), indicating that Dectin-1 mediated signalling plays a more relevant role than TLR2 mediated signalling in the response to *C. albicans*. Pam_2_CSK_4_ appears to promote differentiation to macrophages rather than to moDCs according with previous reports [3], [19]. Accordingly, cells with DC morphology were generated in response to both Curdlan and yeasts, although the morphology of DCs generated in response to Curdlan shows more dendritic extensions than the DCs generated in response to yeasts, whereas these cells were not observed in response to Pam_2_CSK_4_. Therefore, the response to *C. albicans* yeasts that are recognized simultaneously by both receptors [16] is similar but not identical to the response to Curdlan. Although the levels of cytokines (TNF-α and GM-CSF) in the supernatants of cells at day 4 and 7 (either in presence or absence of yeasts) were undetectable (data not shown) and also the TNF-α levels were not detectable when cells were further incubated without stimuli, the possible induction of endogenous putative growth and differentiation factors cannot be completely ruled out. Overall, these results clearly indicate that *C. albicans* may alter mature cell production by promoting the generation of moDCs by a mechanism involving both Dectin-1 and TLR2/MyD88. These *in vitro* results clearly correlate with the *in vivo* results, although the *in vivo* generation of moDCs was dependent on TLR2 whereas *in vitro* was only partially dependent on TLR2. Other authors have already described the involvement of TLRs in DCs development. CLPs from mice with active herpes infection are biased to DC differentiation in a largely TLR9 dependent manner [6]. Moreover, signalling through different TLRs results in differing effects on the *in vitro* production of bone marrow derived DCs induced by GM-CSF. Ligands for TLR4 and TLR9 drive the production of pDCs, whereas ligands for TLR3 and influenza viruses reduce the production of bone marrow derived DCs, resulting in increased neutrophil generation [35].

The role of TLR2/MyD88 in the differentiation of Lin^−^ cells are in line with several previous reports showing that TLRs have a role in haematopoiesis during infection [3], [4], [5], [6], [7], [8], [18], [19], [34], [35]. However, this is the first report showing a possible role for Dectin-1 in the differentiation of progenitor cells. Although Dectin-1 is not expressed on the most primitive stem cells (SP), the expression of this receptor is acquired by progenitors before their differentiation to mature cells, as Lin^−^ cells actually express significant levels of Dectin-1.

Lin^−^ progenitor cells differentiated in the presence of yeasts (moDCs) acquire significant fungicidal activity and are able to secrete TNF-α. Interestingly, these cells produce more TNF-α in response to hyphae than in response to yeasts, suggesting that these cells possess an increased ability to recognize and respond to the more virulent fungal form, which is not recognized by control cells. These cells are phenotypically similar to a recently described subset of DCs, specialized in innate immunity against pathogens, named TipDCs (for TNF-α-iNOS producing DCs) that display a remarkable microbicidal activity and also provide iNOS dependent help for antibody production by B cells [27], [33]. As a conclusion, our results identified a subset of DCs that (i) express the markers of moDCs, (ii) are newly generated during *in vivo* candidiasis in a TLR2 dependent manner, (iii) are generated *in vitro* from Lin^−^ progenitors cells in response to *C. albicans* by a TLR2/MyD88 and Dectin-1 dependent mechanism, (iv) are able to secrete TNF-α, and (v) have fungicidal activity. Therefore, these cells are functional and would be involved in innate immunity against the pathogen, although their possible role in the induction of antigen-specific adaptive immunity has still to be determined. These data support the notion that TLR mediated signalling may lead to reprogramming early progenitors to generate functionally improved phenotypes of mature cells to deal with the pathogen, and therefore the manipulation of this process may help to boost the innate immune response during serious infections.

## Materials and Methods

### Mice

TLR4^−/−^, MyD88^−/−^, and TLR2^−/−^ mice (C57BL/6 background) provided by Dr. Shizuo Akira (Osaka University, Osaka, Japan) were bred and maintained at the animal production service facilities (University of Valencia); wild-type C57BL/6 mice (Harlan Ibérica, Barcelona, Spain) were used as controls. Mice of both sexes between 8 and 12 weeks old were used. Femurs and tibias from control and Dectin-1^−/−^ mice (C57BL/6 background) [36] were kindly provided by Dr. David M. Underhill and Dr. Helen S. Goodridge (Immunobiology Research Institute, Cedars-Sinai Medical Center, CA, USA).

### Ethics Statement

This study was carried out in strict accordance with the recommendations in the “Real Decreto 1201/2005, BOE 252” for the Care and Use of Laboratory Animals of the “Ministerio de la Presidencia”, Spain. The protocol was approved by the Committee on the Ethics of Animal Experiments of the University of Valencia (Permit Number: A1264596506468). All efforts were made to minimize suffering.

### *C. albicans* infection and *in vivo* assays

Cells of *C. albicans* PCA2, a low-virulence non-germinative strain, were obtained as previously described [20], [37] and diluted in PBS to the appropriate cell concentration. Mice were challenged intravenously with 1.5×10^6^ yeasts in a volume of 0.1 ml of PBS. To assess the tissue outgrowth of the microorganism, three mice of each group were killed at days three, seven and fourteen post-infection, and the kidneys, spleen and bone marrow from femurs and tibias were removed aseptically. Kidneys and spleen were weighed, and homogenized in 1 ml of PBS; dilutions of the homogenates were plated on Sabouraud dextrose agar. The CFUs were counted after 24 h of incubation at 37°C and expressed as CFUs per gram of tissue. Whole bone marrow cell suspension was also diluted and plated, and the CFUs were counted and expressed as CFUs per total bone marrow from one animal. Uninfected mice (n = 3) were used as controls (day 0).

To assess the changes in bone marrow and spleen cell populations, three mice of each group were killed at days three, seven and fourteen post-infection, and the spleen and bone marrow from femurs and tibias were removed aseptically. Total spleen cells were obtained by collagenase D treatment of the spleens as previously described [37], [38]. Erythrocytes were lysed in ammonium chloride buffer. For analysis, cells were microscopically counted, labeled with various combinations of antibodies and analyzed by flow cytometry, as described below. Bone marrow and spleen cells from uninfected mice (n = 3) were used as controls (day 0).

### Cell purification and culture

Murine bone marrow was obtained by flushing the femurs and tibias. Cells were depleted of lineage positive cells by immunomagnetic cell sorting using MicroBeads (Miltenyi Biotec, Madrid, Spain). Briefly, bone marrow cells were labelled with a cocktail of antibodies against a panel of lineage antigens [CD5, CD45R (B220), CD11b, Gr-1 (Ly-6G/C), 7-4, and Ter-119], and then cells were purified by negative selection according to the manufacturer's instructions (Lin^−^ progenitor cells). Purity of the sorted cells was assessed by labelling with anti-Lin cocktail and flow cytometry analysis, and no Lin^+^ cells were detected. Purified cells were immediately cultured in complete cell culture medium (RPMI 1640 medium supplemented with 5% heat-inactivated FBS and 1% penicillin-streptomycin stock solution, Gibco, Barcelona, Spain) containing three cytokines: stem cell factor (SCF, 20 ng/ml), Flt-3 ligand (FL, 100 ng/ml) (Peprotech, Rocky Hill, NJ) and IL-7 (10 ng/ml) (MBL, Woburn, MA). Cells were cultured in flat-bottomed 24-well plates at a density of 400,000 cells per well in 0.5 ml and challenged for 7 days with the indicated stimuli. At day four, the culture medium was renovated including the cytokines and the stimuli.

### Antibodies and flow cytometry analyses

The following antibodies used in flow cytometry analyses were purchased from Miltenyi Biotec (Madrid, Spain): cocktail of biotinylated anti-lineage antigens [CD5, CD45R (B220), CD11b, Gr-1 (Ly-6G/C), 7-4, and Ter-119], FITC or APC-labeled anti-Sca-1 (clone D7), RPE-labeled anti-c-Kit (clone 3C1), APC-labeled anti-Gr-1 (clone RB6-8C5) and APC-labeled anti-mPDCA-1 (clone JF05-1C2.4.1). The following antibodies used in flow cytometry analyses were purchased from eBioscience (San Diego, CA): FITC-labeled anti-CD11b (clone M1/70), PE-labeled anti-B220 (clone RA3-6B2), FITC-labeled anti-CD19 (clone 1D3), PE-Cy7-labeled anti-F4/80 (clone BM8), PE-labeled anti-CD11c (clone N418), PE-labeled anti-TLR2 (clone T2.5) and PE-labeled anti-TLR4 (clone MTS510). The following antibodies used in flow cytometry analyses were purchased from BD Pharmingen (San Jose, CA): FITC-labeled anti-c-Kit (clone 2B8), PE-Cy7-labeled anti-IL-7Rα (clone SB-119), PerCP-Cy5.5-labeled anti-Ly6C (clone AL-21), APC-H7-labeled anti-CD4 (clone GK1.5) and APC-labeled anti-CD8α (clone 53-6.7). FITC-labeled anti-Dectin-1 (clone 2A11) monoclonal was purchased from AbD Serotec (Oxford, UK).

To identify the so-called side population (SP) cell staining was performed with the vital dye Hoechst 33342 (Molecular Probes, Invitrogen, Eugene, OR). Briefly, whole bone marrow was isolated, erythrocytes were lysed in ammonium chloride buffer, and cells were resuspended in staining media at 10^6^ cells per ml and incubated with 5 µg/ml Hoechst 33342 for 90 min at 37°C with agitation in the dark. Afterwards, cells were labelled with different antibodies, and analyzed on a MoFlo high-speed sorter (Beckman Coulter), in the presence of propidium iodide at 10 µg/ml [28].

Flow cytometry analyses were performed on a FACSCanto (BD Biosciences) or a MoFlo high-speed sorter (Beckman Coulter), and the data were analyzed with FACSDiva and SUMMIT V4.3.01 software, respectively.

### Photomicrographs

Cultured Lin^−^ progenitor cells were stained with Rapid Panoptic (Panreac Química S.A., Barcelona, Spain), mounted in 50% glycerol in PBS and cell morphology was observed on a Nikon ECLIPSE E800. Photomicrographs were taken with a Nikon DXM1200F camera.

### Determination of *C. albicans* killing by *in vitro* differentiated cells

Lin^−^ progenitor cells from C57BL/6 mice were cultured in presence or absence of inactivated *C. albicans* yeasts, as above described. Afterwards, cells were washed once with PBS and plated in flat-bottomed 96-well plates at a density of 400,000 cells in 200 µl of complete cell culture medium. Cells were challenged with viable PCA2 yeasts at a 1∶3 ratio (murine cell∶yeast), settled onto the cells by centrifugation, and incubated for 1 h. As control *C. albicans* cells were inoculated in culture medium without murine cells. After incubation samples were diluted in water, plated on Sabouraud dextrose agar and incubated overnight at 37°C. The colonies were counted and expressed as percentage of CFUs as compared with CFUs at time 0 h (100%). A non-germinative strain (PCA2) was chosen for killing assays, in order to facilitate determination of CFUs after the incubation period, as no germ tube (hyphae) aggregates are formed. Triplicate samples were analyzed in each assay.

### Measurement of *in vitro* cytokine production

Lin^−^ progenitor cells from C57BL/6 mice were cultured in presence or absence of inactivated *C. albicans* yeasts, as above described. Afterwards, cells were washed once with PBS and plated in flat-bottomed 96-well plates at a density of 250,000 cells in 200 µl of complete cell culture medium. Cells were challenged with the indicated stimuli for 24 h and cell-free supernatants were then harvested and tested for TNF-α release by commercial ELISA kit (eBioscience, San Diego, CA). Duplicate samples were analyzed in each assay.

### Stimuli used and preparation of fungal stimuli

The stimuli used were Ultrapure *E. coli* LPS (Invivogen, San Diego, CA), Pam_2_CSK_4_ (Invivogen), ODN 1585 (Invivogen), Curdlan (Wako Chemicals, Richmond, VA) and two inactivated *C. albicans* ATCC 26555 forms, yeast and hypha, obtained as previously reported [20], [38]. Briefly, starved yeast cells were inoculated (200 µg [dry weight] of cells per ml) in a minimal synthetic medium and incubated for 3 h at 28°C to obtain yeasts or at 37°C to obtain hyphae; more than 90% of the cells exhibited well-defined germ tubes (true hyphae) at 37°C, whereas only yeast cells were observed at 28°C. For inactivation, fungal cells were resuspended (20×10^6^ cells/ml) in 4% paraformaldehyde (fixation buffer, eBioscience, San Diego, CA) and incubated for 1 h at room temperature. After treatment, fungal cells were extensively washed in PBS and brought to the desired cell density in cell culture medium. All procedures were performed under conditions designed to minimize endotoxin contamination as described elsewhere [20], [38].

### Statistical analysis

Statistical differences were determined using Student's two-tailed *t*-test. Data are expressed as mean ± SD. Significance was accepted at *P<0.05 and ** *P*<0.01 level.
